# Custom

**Published:** 1859-11

**Authors:** 


### CUSTOM.

"Custom to whom custom is due," was the conservative precept of Apostolic times, when the law, enacted by the constituted authorities of the country, was the "*higher law*," and *obedience to the powers that be*, was enjoined as one of the first duties of a good citizen, while those who violated these laws, "resisted the ordinance of God." Fanaticism run mad practically advocates the notion now-a-days, that every man has a right to interpret the Scriptures for himself, which is really nothing more or less than making his own judgment a rule of right, and, if thwarted, in acting out that judgment in his relation to others, affords him ground for saying that liberty of conscience is denied him, while those who do not accord with him in his interpretations, are denounced as hater of God's word, and enemies to his religion; and we are precipitated at once into that most terrible of all rules, the anarchy of re-religious fanaticism. As well may every man claim the right to interpret the Constitution of our common country, and act up to his interpretation. The "most direct route" to a peaceful and prosperous government, is for those who do not like it, to clear out, and go to some country whose laws are more congenial. Mexico wants money, and has land to sell, so with the Central American States—a territory for sale, large enough to give a good sized state to each considerable body of fanatics, who, against the peace of our common country, are laboring in so many ways to sap its prosperity, and interfere with its true advancement. Prudent men, thoughtful men, see with pain, how many wild-brained, hot-headed, unbalanced young men, and old too, make a hustings of the pulpit, and goaded on by a "*zeal not according to knowledge*," soon find themselves out of sight of the Cross which they had sworn (and for which they receive pay) to preach; and instead of girding themselves, sailing on a sea of love, winning souls to Christ, are enveloped in storms of passion, and with lowering eyes, and corrugated forehead, and clenched fist, and stamping foot, and thundering voice, hurl forth whole avalanches of bootless wrath; or spitting out seething curses through gritted teeth, their hearts the meanwhile (and from which all these things come) being the very pandemonium of of imprecation and anathema. And against whom? their brother man! and for what? differing from them in opinion! Shame, oh shame, to bosoms so inhuman—a burning shame to heads so intolerant!

These brimstone preachers, who have got into office by mistake, are dyspepsic men, or have been crossed in love or ambition, and should be promptly placed under a "regimen" of bread and water, and cracking rock for the turnpike. It is perfectly wonderful how steady, hard work, and plain food knock insanity and ill health out of a man; and at one and the same time put common sense and good health in their place. The gentleman who amused himself sometime ago with "making paper" and smoking cigars, found they did not agree with him, but reduced his flesh, spoiled his color, and took away his strength, causing constant head-ache, dormant appetite, sleepless nights, and scheming days, and turned right about, dashed away his cigar, emptied his wine-glass, threw down his pen, and, by driving nails, making pine boxes, drinking cold water, and coffee made out of burnt bread crust, with corned beef and stirabout, came to enjoy perfect heath, and gained fifteen pounds of flesh during the first sixty days of his *Sing Sing* sojourn, and no doubt has his wits about him as well as any other man. There is great potency in plain food and hard work, they have wonderful virtue in clearing the cobwebs from the brain of opinionated people. Plain food and hard work! magnificent pills are they! they bring a man down from æriel heights, and sober his views, and make him fit to live like common folks and among common folks, in a time surprisingly short. They did more for the forger in two months than homœopathy accomplished in two years; this we account for, however, in the antagonistic qualities of the prices and pills, the latter were *secundem artem*, really infinitessimal, sweet as sugar, and as easy to take as the mountain-sized fees of the far-seeing doctor. The amount of the whole matter is this, the only way to have sober, true, and just views of men and things, is to live on plain food, and engage in some moderate remunerative work for several hours of every day, by these, with light suppers, regular habits, great personal cleanliness, and, freely associating with intelligent men of all parties and sects, we believe a leaven of strong common sense, of liberality of sentiment, of conservatism and sound morals, would, in no great length of time, pervade this entire land, make it a whole brotherhood of States, and so bind them together, that true progress and solid prosperity would be witnessed in all our borders, the ensignia of a great nation, and of a people happy and free.

